# Supplementary material for: Development and Validation of the Nursing Care and Religious Diversity Scale (NCRDS)
Source: Healthcare (Basel). 2023 Jun 21;11(13):1821. doi: 10.3390/healthcare11131821 (PMC10340361; doi:10.3390/healthcare11131821)
Supplement: Supplementary file 1 [file healthcare-11-01821-s001.zip › healthcare-2378117-supplementary.pdf]

APPENDIX

Table S1. NCRDS item frequencies

		1	2	3	4	5
**NCRDS 1**	Frequency	28	50	132	60	47
	%	8,8	15,8	41,6	18,9	14,8
**NCRDS 2**	Frequency	38	71	100	57	51
	%	12,0	22,4	31,5	18,0	16,1
**NCRDS 3**	Frequency	30	82	119	55	31
	%	9,5	25,9	37,5	17,4	9,8
**NCRDS 4**	Frequency	36	54	115	66	46
	%	11,4	17,0	36,3	20,8	14,5
**NCRDS 5**	Frequency	59	68	118	47	25
	%	18,6	21,5	37,2	14,8	7,9
**NCRDS 6**	Frequency	21	72	133	50	41
	%	6,6	22,7	42,0	15,8	12,9
**NCRDS 7**	Frequency	64	59	40	31	123
	%	20,2	18,6	12,6	9,8	38,8
**NCRDS 8**	Frequency	34	36	49	45	153
	%	10,7	11,4	15,5	14,2	48,3
**NCRDS 9**	Frequency	53	74	77	51	62
	%	16,7	23,3	24,3	16,1	19,6
**NCRDS 10**	Frequency	80	73	76	43	45
	%	25,2	23,0	24,0	13,6	14,2
**NCRDS 11**	Frequency	108	92	58	35	24
	%	34,1	29,0	18,3	11,0	7,6
**NCRDS 12**	Frequency	31	39	40	49	158
	%	9,8	12,3	12,6	15,5	49,8
**NCRDS 13**	Frequency	88	119	85	19	6
	%	27,8	37,5	26,8	6,0	1,9
**NCRDS 14**	Frequency	81	111	101	16	8
	%	25,6	35,0	31,9	5,0	2,5
**NCRDS 15**	Frequency	93	102	79	28	15
	%	29,3	32,2	24,9	8,8	4,7
**NCRDS 16**	Frequency	183	48	52	25	9
	%	57,7	15,1	16,4	7,9	2,8
**NCRDS 17**	Frequency	32	83	144	35	23
	%	10,1	26,2	45,4	11,0	7,3
**NCRDS 18**	Frequency	20	65	132	57	43
	%	6,3	20,5	41,6	18,0	13,6
**NCRDS 19**	Frequency	31	69	137	49	31
	%	9,8	21,8	43,2	15,5	9,8
**NCRDS 20**	Frequency	29	55	122	57	54
	%	9,1	17,4	38,5	18,0	17,0

**NCRDS 21**	Frequency	36	101	108	55	17
	%	11,4	31,9	34,1	17,4	5,4
**NCRDS 22**	Frequency	57	55	115	62	28
	%	18,0	17,4	36,3	19,6	8,8
**NCRDS 23**	Frequency	38	105	123	31	20
	%	12,0	33,1	38,8	9,8	6,3
**NCRDS 24**	Frequency	19	47	130	79	42
	%	6,0	14,8	41,0	24,9	13,2
**NCRDS 25**	Frequency	45	83	114	52	23
	%	14,2	26,2	36,0	16,4	7,3

Table S2. KMO and Bartlett test[at]

Kaiser-Meyer-Olkin measure of sampling adequacy.		.780
Bartlett sphericity test	Approx. Chi-square	4805.085
	Gl	528
	Sign.	.000

a. Based on correlations

Table S3. Commonality

	Crude		Modified scale	
	Initial	Extraction	Initial	Extraction
NCRDS_1	1.739	1.078	1.000	.620
NCRDS_2	.971	.328	1.000	.338
NCRDS_3	.620	.098	1.000	.159
NCRDS_4	2.571	2.305	1.000	.896
NCRDS_5	2.014	1.654	1.000	.821
NCRDS_6	1.975	1.360	1.000	.689
NCRDS_7	1.851	1.288	1.000	.696
NCRDS_8	1.849	1.394	1.000	.754
NCRDS_9	1.567	1.059	1.000	.676
NCRDS_10	1.608	1.057	1.000	.657
NCRDS_11	1.232	.759	1.000	.617
NCRDS_12	1.110	.328	1.000	.295
NCRDS_13	1.094	.226	1.000	.207
NCRDS_14	1.044	.491	1.000	.470
NCRDS_15	1.420	.942	1.000	.663
NCRDS_16	1.168	.530	1.000	.454
NCRDS_17	1.026	.639	1.000	.623
NCRDS_18	1.184	.696	1.000	.588
NCRDS_19	1.169	.789	1.000	.675
NCRDS_20	1.378	.952	1.000	.691
NCRDS_21	9.352	9.332	1.000	.998
NCRDS_22	1.348	.582	1.000	.431
NCRDS_23	1.230	.762	1.000	.619
NCRDS_24	1.172	.809	1.000	.691
NCRDS_25	1.199	.661	1.000	.551
NCRDS_26	1.530	1.051	1.000	.687
NCRDS_27	1.274	.945	1.000	.741

NCRDS_28	1.407	.844	1.000	.599
NCRDS_29	1.323	.726	1.000	.548
NCRDS_30	.949	.650	1.000	.685
NCRDS_31	.931	.707	1.000	.760
NCRDS_32	1.250	1.008	1.000	.806
NCRDS_33	1.287	.920	1.000	.715

Extraction method: Analysis of the main components.

Table S4. Total variance explained

		Initial eigenvalues[to]			Loading sums of extraction squares			Loading of rotation squares sums		
	Component	Total	% variance	% cumulative	Total	% variance	% cumulative	Total	% variance	% cumulative
Crude	1	10.022	18.965	18.965	10.022	18.965	18.965	5.361	10.145	10.145
	2	8.271	15.652	34.617	8.271	15.652	34.617	3.538	6.696	16.841
	3	5.620	10.636	45.252	5.620	10.636	45.252	3.757	7.109	23.950
	4	4.365	8.260	53.512	4.365	8.260	53.512	3.477	6.580	30.530
	5	2.757	5.218	58.730	2.757	5.218	58.730	4.516	8.546	39.076
	6	2.489	4.710	63.439	2.489	4.710	63.439	4.427	8.378	47.454
	7	1.814	3.433	66.873	1.814	3.433	66.873	2.732	5.170	52.624
	8	1.633	3.090	69.963	1.633	3.090	69.963	9.163	17.339	69.963
	9	1.505	2.849	72.812						
	10	1.293	2.447	75.259						
	11	1.241	2.348	77.607						
	12	1.136	2.150	79.758						
	13	1.055	1.996	81.753						
	14	.950	1.798	83.551						
	15	.846	1.600	85.151						
	16	.750	1.420	86.571						
	17	.698	1.321	87.892						
	18	.657	1.243	89.135						
	19	.585	1.108	90.242						
	20	.559	1.058	91.301						
	21	.523	.989	92.290						
	22	.495	.936	93.226						
	23	.485	.918	94.144						
	24	.459	.869	95.013						
	25	.413	.781	95.795						
	26	.374	.707	96.502						
	27	.346	.656	97.157						
	28	.311	.589	97.746						
	29	.296	.559	98.306						
	30	.277	.524	98.830						
	31	.234	.442	99.272						
	32	.214	.404	99.676						
	33	.171	.324	100.000						
Modified scale	1	10.022	18.965	18.965	1.953	5.919	5.919	4.101	12.426	12.426
	2	8.271	15.652	34.617	5.369	16.270	22.189	3.048	9.235	21.661
	3	5.620	10.636	45.252	3.613	10.948	33.137	2.998	9.086	30.747
	4	4.365	8.260	53.512	3.343	10.129	43.266	2.651	8.033	38.781
	5	2.757	5.218	58.730	1.960	5.940	49.207	2.622	7.947	46.727
	6	2.489	4.710	63.439	1.519	4.604	53.811	2.053	6.222	52.949
	7	1.814	3.433	66.873	1.396	4.232	58.043	1.814	5.496	58.445
	8	1.633	3.090	69.963	1.267	3.840	61.882	1.134	3.438	61.882
	9	1.505	2.849	72.812						
	10	1.293	2.447	75.259						

	11	1.241	2.348	77.607						
	12	1.136	2.150	79.758						
	13	1.055	1.996	81.753						
	14	.950	1.798	83.551						
	15	.846	1.600	85.151						
	16	.750	1.420	86.571						
	17	.698	1.321	87.892						
	18	.657	1.243	89.135						
	19	.585	1.108	90.242						
	20	.559	1.058	91.301						
	21	.523	.989	92.290						
	22	.495	.936	93.226						
	23	.485	.918	94.144						
	24	.459	.869	95.013						
	25	.413	.781	95.795						
	26	.374	.707	96.502						
	27	.346	.656	97.157						
	28	.311	.589	97.746						
	29	.296	.559	98.306						
	30	.277	.524	98.830						
	31	.234	.442	99.272						
	32	.214	.404	99.676						
	33	.171	.324	100.000						

Extraction method: Analysis of the main components.
a. When analyzing a covariance matrix. the initial eigenvalues are the same in the raw and modified scale solution.

Table S5. Component array[a]

	Crude Component								Modified scale Component							
	1	2	3	4	5	6	7	8	1	2	3	4	5	6	7	8
NCRDS_21	-2.943	.718	.107	.123	-.310	-.179	-.011	-.048	-.962	.235	.035	.040	-.101	-.058	-.004	-.016
NCRDS_13	.289	.170	-.089	.174	-.004	.023	.192	-.196	.276	.163	-.085	.166	-.004	.022	.184	-.187
NCRDS_4	.221	.931	-.741	-.432	.452	-.606	.242	.154	.138	.581	-.462	-.269	.282	-.378	.151	.096
NCRDS_25	.302	.620	.253	.144	-.117	-.114	-.097	-.255	.276	.566	.231	.131	-.107	-.104	-.089	-.233
NCRDS_19	-.103	.594	.364	-.029	.127	.255	.456	-.055	-.095	.550	.337	-.027	.118	.236	.421	-.050
NCRDS_23	.205	.597	.482	.170	-.084	-.172	-.205	-.156	.185	.538	.434	.153	-.076	-.155	-.184	-.141
NCRDS_24	.220	.559	.433	.151	.045	-.233	-.410	-.115	.203	.517	.400	.140	.041	-.215	-.379	-.106
NCRDS_28	.413	.608	.426	-.263	-.182	-.004	-.091	-.106	.349	.512	.359	-.221	-.153	-.003	-.077	-.089
NCRDS_9	.106	.616	-.596	-.103	-.136	.494	-.020	-.200	.085	.492	-.476	-.083	-.109	.395	-.016	-.160
NCRDS_20	.172	.557	.433	-.425	-.107	.197	.431	-.095	.146	.475	.369	-.362	-.091	.168	.367	-.081
NCRDS_18	.037	.487	.449	-.176	.080	.358	.299	.025	.034	.448	.413	-.162	.073	.329	.275	.023
NCRDS_16	.179	.449	.373	-.281	.112	.127	.085	.205	.166	.416	.345	-.260	.104	.117	.079	.189
NCRDS_12	.223	.411	-.030	.086	-.087	-.184	.057	-.236	.212	.390	-.029	.081	-.083	-.174	.054	-.224
NCRDS_26	.125	.603	.714	-.184	.144	.085	-.290	.130	.101	.487	.577	-.148	.116	.069	-.234	.105
NCRDS_27	.207	.575	.648	-.059	.077	.013	-.372	-.057	.183	.510	.574	-.052	.068	.012	-.329	-.051
NCRDS_8	.145	.680	-.763	.138	-.142	.440	-.304	-.050	.107	.500	-.561	.101	-.104	.324	-.223	-.037
NCRDS_7	.171	.618	-.721	-.073	-.357	.336	-.333	-.031	.126	.454	-.530	-.054	-.262	.247	-.245	-.023
NCRDS_5	-.022	.712	-.743	-.082	.567	-.494	.034	.146	-.016	.502	-.523	-.058	.399	-.348	.024	.103
NCRDS_22	-.033	.234	.481	.364	.299	-.080	-.251	.057	-.029	.202	.414	.313	.258	-.069	-.216	.049
NCRDS_32	.132	.188	-.068	.844	-.190	-.072	.037	.441	.118	.168	-.061	.755	-.170	-.065	.033	.395
NCRDS_31	.235	.248	-.051	.601	-.316	-.063	.098	.336	.244	.257	-.053	.623	-.327	-.066	.102	.349
NCRDS_6	.062	.657	-.304	-.838	-.152	-.109	-.248	.181	.044	.468	-.216	-.596	-.108	-.078	-.176	.129
NCRDS_30	.256	.206	-.083	.568	-.329	-.131	.053	.289	.263	.211	-.085	.584	-.338	-.134	.054	.297
NCRDS_15	.209	.545	-.110	.675	.048	-.131	.151	-.301	.176	.457	-.092	.567	.040	-.110	.127	-.253
NCRDS_11	.022	.349	.034	.625	.477	-.012	.083	-.098	.020	.315	.031	.563	.430	-.011	.075	-.088
NCRDS_14	.067	.306	.093	.410	.405	.124	.090	-.169	.066	.299	.091	.401	.396	.121	.089	-.166

NCRDS_33	.354	.479	-.169	.128	-.629	-.060	.253	.239	.312	.422	-.149	.113	-.554	-.053	.223	.211
NCRDS_1	-.467	.012	.041	-.235	.583	.477	-.004	.485	-.354	.009	.031	-.179	.442	.361	-.003	.368
NCRDS_2	-.144	-.018	-.058	.309	.435	.012	.026	-.133	-.146	-.019	-.059	.314	.442	.013	.027	-.135
NCRDS_3	-.002	.014	-.098	.176	.211	.055	-.079	.062	-.003	.018	-.124	.223	.268	.070	-.101	.079
NCRDS_10	-.233	.127	-.412	.399	.327	.705	-.229	.000	-.184	.100	-.325	.315	.258	.556	-.181	.000
NCRDS_17	.014	.511	.104	.026	-.080	.308	.514	-.023	.014	.505	.103	.026	-.079	.304	.507	-.022
NCRDS_29	.152	.303	.451	.089	.004	.060	-.084	.623	.132	.263	.392	.077	.004	.053	-.073	.542

Extraction method: Analysis of the main components.
a. 8 extracted components.

Table S6. Matrix of rotated components[to]

	Crude Component								Modified scale Component							
	1	2	3	4	5	6	7	8	1	2	3	4	5	6	7	8
NCRDS_27	.943	-.043	.222	.031	.009	-.040	-.011	-.034	.835	-.038	.197	.028	.008	-.035	-.009	-.030
NCRDS_24	.859	.110	-.018	.120	.009	.093	.189	.022	.793	.101	-.016	.111	.008	.086	.175	.020
NCRDS_26	.925	-.058	.368	-.014	-.042	.015	-.232	-.012	.748	-.047	.298	-.011	-.034	.012	-.187	-.010
NCRDS_23	.781	.175	.178	.063	-.010	.019	.286	.062	.704	.158	.161	.057	-.009	.017	.258	.056
NCRDS_28	.713	.006	.392	-.277	.120	.086	.213	-.194	.601	.005	.330	-.233	.101	.073	.179	-.163
NCRDS_25	.612	.162	.219	.057	.152	.089	.421	-.032	.559	.148	.200	.052	.139	.081	.384	-.029
NCRDS_22	.558	.128	-.014	.423	-.216	-.086	-.110	.097	.480	.110	-.012	.364	-.186	-.074	-.095	.083
NCRDS_29	.479	.442	.234	-.052	-.141	.019	-.461	-.102	.417	.384	.204	-.045	-.123	.017	-.401	-.089
NCRDS_31	.071	.820	.026	.090	.081	-.023	.115	-.040	.074	.849	.027	.093	.084	-.024	.119	-.042
NCRDS_32	.040	.945	-.095	.316	.031	-.054	.003	.033	.036	.845	-.085	.282	.027	-.049	.003	.030
NCRDS_30	.069	.778	-.056	.052	.070	-.001	.164	-.054	.071	.798	-.057	.053	.071	-.001	.169	-.056
NCRDS_33	.060	.686	.289	-.396	.291	.166	.293	-.080	.053	.605	.255	-.349	.257	.146	.259	-.071
NCRDS_19	.240	.007	.817	.186	.007	.059	.030	.156	.222	.006	.756	.172	.006	.055	.028	.145
NCRDS_17	.014	.168	.746	.067	.174	.036	.120	.059	.014	.166	.737	.066	.171	.036	.118	.059
NCRDS_20	.336	-.129	.851	-.267	.006	.081	.123	-.078	.286	-.110	.725	-.228	.005	.069	.105	-.066
NCRDS_18	.331	-.058	.753	.029	.035	-.052	-.107	-.017	.304	-.054	.692	.026	.032	-.048	-.098	-.016
NCRDS_16	.438	-.017	.490	-.101	-.011	.159	-.202	-.145	.406	-.016	.453	-.094	-.010	.147	-.187	-.134
NCRDS_11	.180	.237	.114	.790	-.007	.119	.136	.032	.162	.213	.103	.712	-.006	.107	.123	.029
NCRDS_14	.182	.069	.210	.624	.046	.023	.121	-.043	.179	.067	.206	.611	.045	.022	.118	-.042
NCRDS_2	-.073	-.078	-.056	.551	-.063	.037	.001	.069	-.074	-.079	-.056	.559	-.064	.038	.001	.070
NCRDS_3	-.011	.051	-.087	.265	.065	.044	-.102	-.038	-.014	.065	-.111	.336	.083	.056	-.129	-.048
NCRDS_8	.036	.224	.006	.142	1.117	.259	.093	-.012	.027	.164	.005	.104	.821	.190	.068	-.009
NCRDS_7	.057	.186	-.034	-.177	1.065	.259	.124	-.006	.042	.137	-.025	-.130	.783	.191	.091	-.004
NCRDS_9	-.058	-.012	.295	.026	.949	.201	.164	-.022	-.047	-.009	.236	.021	.758	.160	.131	-.018
NCRDS_10	-.186	.006	-.021	.643	.675	-.202	-.323	.087	-.147	.004	-.016	.507	.532	-.160	-.254	.068
NCRDS_4	.063	.001	.214	.043	.318	1.453	.187	-.083	.040	.001	.133	.027	.198	.906	.117	-.052
NCRDS_5	-.005	.042	-.052	.362	.314	1.187	.051	.092	-.004	.029	-.037	.255	.221	.836	.036	.065
NCRDS_6	.331	-.242	.146	-.624	.515	.702	-.149	.040	.235	-.172	.104	-.444	.366	.500	-.106	.028
NCRDS_1	-.112	-.270	.264	.297	.058	.088	-.895	.148	-.085	-.205	.200	.225	.044	.067	-.679	.112
NCRDS_15	.203	.438	.141	.541	.188	.139	.583	.041	.170	.367	.118	.454	.157	.117	.489	.034
NCRDS_12	.242	.115	.127	.030	.125	.208	.424	-.029	.229	.109	.120	.029	.119	.197	.402	-.028
NCRDS_13	-.008	.124	.159	.146	.106	.042	.332	-.203	-.008	.118	.152	.140	.101	.040	.317	-.194
NCRDS_21	-.136	-.237	.272	.168	.052	.073	-.464	2.989	-.044	-.078	.089	.055	.017	.024	-.152	.977

Extraction method: Analysis of the main components.
Rotation method: Varimax with Kaiser normalization.
a. Convergence for rotation performed in 7 iterations.

Table S7. Component Transformation Matrix

Component	1	2	3	4	5	6	7	8
1	.204	.193	-.005	-.091	.054	.048	.263	-.916
2	.532	.193	.461	.099	.424	.449	.141	.236
3	.599	-.059	.313	-.044	-.577	-.440	-.099	.038
4	-.015	.642	-.188	.633	-.031	-.302	.213	.114
5	.041	-.407	.014	.722	-.198	.356	-.306	-.229
6	-.130	-.125	.398	.153	.560	-.561	-.358	-.172
7	-.545	.145	.704	.025	-.340	.128	.228	-.044
8	-.032	.554	.009	-.184	-.123	.231	-.763	-.086

Extraction method: Analysis of the main components.
Rotation method: Varimax with Kaiser normalization.

Table S8. Normality test (n=317)

	Kolmogorov-Smirnov[a]	Sign.
NCRDS_1	0.152	
NCRDS_2	0.482	
NCRDS_3	0.455	
NCRDS_4	0.246	
NCRDS_5	0.288	
NCRDS_6	0.295	
NCRDS_7	0.166	
NCRDS_8	0.175	
NCRDS_9	0.223	
NCRDS_10	0.196	
NCRDS_11	0.181	
NCRDS_12	0.211	
NCRDS_13	0.191	
NCRDS_14	0.206	
NCRDS_15	0.200	
NCRDS_16	0.234	
NCRDS_17	0.236	0.000
NCRDS_18	0.225	
NCRDS_19	0.229	
NCRDS_20	0.205	
NCRDS_21	0.218	
NCRDS_22	0.195	
NCRDS_23	0.227	
NCRDS_24	0.222	
NCRDS_25	0.200	
NCRDS_26	0.172	
NCRDS_27	0.216	
NCRDS_28	0.182	
NCRDS_29	0.194	
NCRDS_30	0.203	
NCRDS_31	0.222	
NCRDS_32	0.212	
NCRDS_33	0.345	

a. Correction of significance of Lilliefors
